# Microenvironment of Sarcomas Associated and Not Associated with Inoculation Sites in Domestic Cats: The Role of Metalloproteinases and Angiogenic Factors

**DOI:** 10.3390/biology15151322

**Published:** 2026-08-06

**Authors:** Carolina Wright, Alejo Carlos Scarano, José Manuel Verdes, Silvana Graciela Scenna, Adriana Graciela Duchene, Juan Manuel Osacar, Lucas Martín Di Pierro, Paula Risso, Erika Badura, Claudio Gustavo Barbeito, Francisco Acuña, Olga Andrea Santelices Iglesias

**Affiliations:** 1 Instituto de Morfología y Patología Animal (IMPA), Facultad de Ciencias Veterinarias, Universidad Nacional de La Plata, La Plata 1900, Buenos Aires, Argentinafacuna@fcv.unlp.edu.ar (F.A.); asantelices@fcv.unlp.edu.ar (O.A.S.I.); 2 Departamento de Patobiología de la Facultad de Veterinaria, Universidad de la República Uruguay, Montevideo 13000, Uruguay; jose.verdes@fvet.edu.uy; 3 Independent Researcher, La Plata 1900, Buenos Aires, Argentina; 4 Cátedra Bioestadística, Facultad de Ciencias Veterinarias, Universidad Nacional de La Plata, La Plata 1900, Buenos Aires, Argentina; 5 Laboratorio de Patología de Aves y Pilíferos, Facultad de Ciencias Veterinarias, Universidad Nacional de La Plata, La Plata 1900, Buenos Aires, Argentina

**Keywords:** soft tissue sarcomas, collagenases, vascularization, ISS, NISS

## Abstract

Soft tissue sarcomas are malignant neoplasms originating from mesenchymal cells. In cats, there are two groups: inoculation site-associated sarcomas (ISSs) and non-inoculation site-associated sarcomas (NISSs). The aim of this study was to investigate the immunoexpression of metalloproteinases (MMPs) 2 and 9, tissue inhibitors (TIMPs) 1 and 2, and the main molecules related to angiogenesis and vascular count in both sarcoma groups; compare their expression; and evaluate their association with angiogenesis. Both tumor groups expressed all the molecules evaluated. A positive correlation was detected between MMP-2 and angiogenic factors in both ISSs and NISSs. MMP-9 expression correlated positively with MMP-2 in both groups. TIMP-1 correlated with both MMP and with angiogenic factors in ISS. Fibroblast growth factor receptor 2 (FGFR-2) expression was higher in NISS. Studying the pathways that regulate angiogenesis in these neoplasms is crucial for identifying ways to intervene and limit neoplastic progression, thereby developing new therapeutic approaches.

## Simple Summary

Soft tissue sarcomas are malignant tumors. In cats, they are classified into two groups: inoculation site-associated sarcomas and non-inoculation site-associated sarcomas. Inoculation site-associated sarcomas are diagnosed in younger animals. They are more aggressive and recurrent than non-inoculation site-associated sarcomas. Studying the aggressiveness of sarcoma behavior requires examining molecules related to tumor growth and the development of new blood vessels from pre-existing ones (angiogenesis). In this study, various molecules that degrade the extracellular matrix (metalloproteinases and their inhibitors) and growth factors with their growth receptors (related to angiogenesis) were analyzed using immunohistochemistry. Image analysis and statistical studies were performed. Both tumor groups expressed all the evaluated molecules. Fibroblast growth factor receptor 2 expression was the only variable that showed significant differences, with significantly higher values in sarcomas not associated with inoculation sites. The results of this study could represent the starting point for new therapeutic approaches based on the specific inhibition of receptors.

## 1. Introduction

Soft tissue sarcomas are malignant neoplasms originating from mesenchymal cells. These tumors can arise in various anatomical locations and exhibit different cellular origins [1,2,3]. However, because both their histological characteristics and biological behavior are similar, they are often studied together [4,5].

Among the sarcomas that affect cats, there is a distinct group of tumors known as injection-site sarcomas (ISSs) [6]. These sarcomas develop at anatomical sites commonly used for the administration of vaccines or other injectable substances, including the interscapular region, lateral thoracic wall, abdominal wall, lumbar region, and hind limbs [7,8]. Sarcomas that arise in anatomical locations not typically used for substance inoculation are classified as non-injection-site sarcomas (NISSs) [5].

Although it is difficult to differentiate between these two groups of tumors, ISSs have some distinctive characteristics. ISSs appear at younger ages, are more aggressive, and have a higher recurrence rate [5,7,8,9,10]. Fibrosarcoma is among the most frequently diagnosed skin and soft tissue tumors in domestic cats, accounting for 80% to 92% of cases [3].

Understanding the aggressive behavior of sarcomas in domestic cats requires studying molecules related to tissue degradation and tumor progression. Matrix metalloproteinases (MMPs) are a family of zinc-dependent endopeptidases that participate in the degradation of the extracellular matrix (ECM) and facilitate tumor invasion [11]. MMPs play a fundamental role in the tumor microenvironment, as they promote the migration and invasion of neoplastic cells, tumor growth, and angiogenesis [8,12,13].

MMP-9 and MMP-2 are gelatinases that degrade type IV collagen and gelatin substrates [13]. High expression of MMP-2 and MMP-9 is associated with tumor cell invasion and migration, and greater metastatic potential in several human neoplasms, such as carcinomas of the breast, colon, esophagus, and stomach [8,13,14].

Under normal conditions, the proteolytic activity of MMP is regulated by endogenous protein inhibitors known as tissue inhibitors of metalloproteinases (TIMPs). Alterations in the MMP-TIMP balance lead to changes in pathophysiological processes, resulting in various diseases such as arthritis, cardiovascular disorders, and emphysema. Furthermore, this imbalance promotes the invasiveness of certain neoplasms and metastasis [15,16]. TIMP also possess biological activities independent of MMP inhibition, such as cell proliferation, inhibition of apoptosis, cell migration and invasion, and angiogenesis [17]. For example, TIMP-1 expression also increases in response to noxious and inflammatory stimuli [18].

Within the TIMP, TIMP-1 and TIMP-2 are inhibitors of MMP-9 and MMP-2 and participate in the regulation of tumor growth, invasion and metastasis, although they have also been described as promoting carcinogenesis through growth-stimulating and anti-apoptotic mechanisms [13,19].

In domestic animals, the expression of MMP and their TIMP has been shown to contribute to invasion and metastasis in mammary tumors. Elevated levels of MMP-2 and MMP-9 have been recorded in these tumors, and MMP-2 expression has been linked to tumor progression, acting as an indicator of tumor growth and invasion [20]. Few studies have evaluated MMP-2 and MMP-9 expression levels in ISSs and NISSs [8,14,21], and those that did either did not include comparative studies or had very small sample sizes.

Given the role that MMPs play in tumor invasiveness mechanisms, they are considered an important area of study in both human and veterinary medicine. This is due to their potential prognostic value and their possible application as therapeutic targets in various types of neoplasms, including sarcomas [14].

MMPs actively participate in angiogenesis by promoting the migration of endothelial cells and the release of vascular endothelial growth factor (VEGF) and other angiogenic factors such as fibroblast growth factor 2 (FGF-2) and transforming growth factor beta (TGF-β) from the ECM in which they accumulate. When released, these factors stimulate the proliferation and migration of endothelial cells [17,22].

Neoplastic growth requires conditions that define the tumor microenvironment. Among these is angiogenesis; that is, the development of new blood vessels from pre-existing blood vessels [23,24]. Among the most relevant factors involved in angiogenesis are VEGF and other molecules that positively regulate this process, including platelet-derived growth factor (PDGF) and FGF, among others [5,25,26,27].

Vascular endothelial growth factor A (VEGF-A) is the most important regulator of angiogenesis [28]. The binding of VEGF to its receptor VEGFR-2 is responsible for the main effects of this signaling pathway, whereas binding to VEGFR-1 results in ligand sequestration and therefore modulates its signaling activity [29]. Both receptors are central regulators of both physiological and pathological angiogenesis [5,30]. Studies on highly invasive neoplasms such as fibrosarcomas in both cats and humans have demonstrated that a significant increase in vascularization correlates with a higher degree of malignancy [31].

PDGF and FGF-2 activate receptors located on the cell surface. Both growth factors are co-expressed in mesenchymal tumors, and their expression is associated with tumor progression and metastatic potential. FGF-2 is closely involved in inflammatory processes, angiogenesis, and the development of tumors of mesenchymal origin [26]. Overexpression of FGF promotes angiogenesis by inducing the secretion of MMP and, through its synergistic action with VEGF, enhances the growth and maturation of tumor blood vessels [27,32].

PDGF is involved in the pathogenesis of various tumor types, including leukemias, gliomas, sarcomas, and carcinomas in humans, by influencing tumor growth, angiogenesis, local invasiveness, and metastasis [26]. This growth factor promotes angiogenesis by upregulating VEGF production, modulating the proliferation and recruitment of perivascular cells, and stabilizing neovascularization through the promotion of blood vessel maturation [27,33].

The aim of this study was to evaluate the immunoexpression of MMP-2, MMP-9, their inhibitors TIMP-1 and TIMP-2, the main effector molecules associated with angiogenesis (VEGF-A, VEGFR-2, PDGFRα, and FGFR-2), and the number of blood vessels immunolabeled with an anti-CD31 antibody in ISSs and NISSs. Additionally, the study compared the expression patterns of the evaluated molecules between both groups of neoplasms and assessed their association with angiogenesis.

## 2. Materials and Methods

### 2.1. Immunohistochemical Studies

This study was approved by the bioethics committee of the Facultad de Ciencias Veterinarias, Universidad Nacional de La Plata (code: 128-3-22T, CICUAL-Institutional Committee for the Care and Use of Laboratory Animals).

Twenty archival cases diagnosed as ISSs (fibrosarcomas) and twenty diagnosed as NISS (fibrosarcomas) were selected (Table 1). A consecutive sampling method was used [34] accepting cases that met the inclusion and exclusion criteria during 2011 and 2012. The criteria for selecting ISS were as follows: 1—inclusion criterion: tumors diagnosed as ISS, particularly fibrosarcoma, during 2011 and 2012; 2—exclusion criterion: cases with typical histological characteristics but atypical (sites not routinely used for substance inoculation) or unknown location were excluded.

For NISSs, the inclusion criteria were as follows: 1—tumors diagnosed as soft tissue sarcomas in the skin and subcutaneous tissue, particularly fibrosarcoma; 2—located in anatomical sites not routinely used for substance inoculation. The exclusion criterion was anatomical location not reported by the referring physician. The sample consisted of 20 cases of ISS and 20 cases of NISS.

Sections of 3 μm from each case were mounted on positively charged slides (HistoSystem, Rosario, Argentina). The sections were then deparaffinized, rehydrated through a graded series of decreasing ethanol (Biopack Productos Químicos, Zárate, Buenos Aires, Argentina) concentrations, and incubated in methanol containing 3% H_2_O_2_ (Biopack Productos Químicos, Zárate, Buenos Aires, Argentina) for 30 min at room temperature to inhibit endogenous peroxidase activity. Subsequently, the slides were washed with phosphate-buffered saline (PBS; pH 7.4, prepared in our laboratory from Sigma-Aldrich salts, St. Louis, MO, USA). Non-specific binding sites were blocked by incubation with 1% bovine serum albumin (Sigma-Aldrich Inc., St. Louis, MO, USA) for 30 min at room temperature in a humid chamber. The samples were then incubated, overnight at 4 °C in a humidified chamber, with each of the primary antibodies listed in Table 2.

Positive controls consisted of feline organs: placenta for MMP-2 and MMP-9, cerebellum for TIMP-1 and TIMP-2, small intestine for VEGF-A, VEGFR-2 and CD31, stomach for FGFR-2, and kidney for PDGFR-α. The same organs used as positive controls were employed as negative antibody controls by replacing each primary antibody with PBS. In addition, non-neoplastic tissues adjacent to the tumors that did not express the antigens of interest were used as internal negative controls. Sections were incubated overnight at 4 °C in a humidified chamber.

Antigen detection was performed using the LSAB system (LSAB Ready-to-Use Kit, BioGenex, HK340, San Ramon, CA, USA) (Table 2), which consisted of incubation for 30 min in a humid chamber at room temperature with a biotinylated dual anti-rabbit/anti-mouse secondary antibody followed by streptavidin–peroxidase (Vector Laboratories, Inc, Newark, CA, USA) and incubation for 30 min in a humid chamber at room temperature. 3,3′-Diaminobenzidine tetrachloride (DAB) (Vector, ZL0918, Newark, NJ, USA) was used as a chromogen and Harris hematoxylin (Biopack, Code 0832.07, Zárate, Buenos Aires, Argentina) as a counterstain. Finally, the samples were dehydrated using a graded series of ethanol, cleared with xylene, and mounted with natural balsam.

### 2.2. Percentage of Identity of the Proteins of Interest

Primary antibodies (anti-VEGF-A, VEGFR-2, PDGFR-α, FGFR-2, and CD-31) previously validated in this species were used [26,35,36]. The remaining antibodies employed in this study were originally developed for use in other species. Therefore, in addition to the controls performed on tissues from the study species, the percentage identity of the proteins of interest between the species for which the antibodies were produced and the study species, *Felis catus*, was evaluated using the Basic Local Alignment Search Tool (BLAST^®^) (https://www.ncbi.nlm.nih.gov/protein/, accessed on 20 June 2026). A sequence identity of ≥70% is considered indicative of protein conservation across species [37,38,39]. High to very high similarity was observed for most of the molecules analyzed between *F. catus* proteins and those of the species for which the antibodies had been developed [40], supporting their suitability and reliability for use in the present study. Because archival material was used, fresh biological material was not available to perform antibody validation by Western blot.

### 2.3. Image Analysis

Images were captured using an analog video camera Sony DXC-390 (Sony, Tokyo, Japan, mounted on a trinocular microscope (CX31, Olympus, Tokyo, Japan) and connected to a computer. The images were subsequently processed using a digital image analysis system (Image-Pro Plus v6.3, Media Cybernetics, Rockville, MD, USA). Immunolabeling for each primary antibody was assessed qualitatively as either positive or negative and quantitatively by calculating the percentage of neoplastic cells exhibiting positive cytoplasmic staining in images obtained from 10 fields at 40× magnification. Immunolabeling of vascular cells for FGFR-2, PDGFR-α, VEGF-A, and VEGFR-2 was evaluated qualitatively only. To determine the total number of vessels (TNVs), blood vessels were counted in 10 fields at 20× magnification in sections stained with the anti-CD31 antibody. The TNVs was determined using ImageJ software (Image-Pro Plus v6.3, Media Cybernetics, Rockville, MD, USA). A single-blind evaluation was performed. The researcher was unaware of the group assignment for each image at the time of the evaluation.

### 2.4. Statistical Analysis

All analyses were performed in R (version 4.5.3) (*n* = 40). The following variables were recorded for each of the 40 cases (20 ISSs and 20 NISSs).

Qualitative variables: tumor group (ISS/NISS); anatomical location of the tumor (Table 1); and, for each of the eight immunohistochemical markers evaluated (MMP-2, MMP-9, TIMP-1, TIMP-2, VEGF-A, VEGFR-2, FGFR-2 and PDGFR-α), positive or negative expression (a case was classified as positive when the percentage of immunolabeled neoplastic cells was greater than 0%).

Quantitative variables: for each of the eight markers, the proportion of cells showing positive cytoplasmic immunolabeling and the total number of vessels (TNVs) were obtained by counting CD31-immunolabeled blood vessels. The complete case-by-case dataset is provided as Supplementary Material S1.

Qualitative variables were compared between the two tumor groups (ISS and NISS).

Fisher’s exact test was used to analyze 2 × 2 contingency tables (group × positive/negative expression). The odds ratio (OR) with a 95% confidence interval was reported as a measure of effect size. Fisher’s exact test was used instead of the chi-square test because the expected frequencies in several cells were less than 5, a condition under which the chi-square test is invalid. The correction for multiple comparisons was applied using the Benjamini–Hochberg (FDR) method.

Differences between groups in the quantitative variables were additionally evaluated from a classical perspective using two different methods: a Welch’s *t*-test, which compares group means without assuming equality of variances and the Mann–Whitney U test as its distribution-free counterpart. Welch’s correction was applied because between-group variances were markedly unequal for several variables. Both tests were applied to the complete set of 40 cases, and the resulting *p*-values were corrected for multiple comparisons using the Benjamini–Hochberg (FDR) procedure. Reporting both a parametric and a non-parametric test allows the robustness of each comparison to be judged independently of distributional assumptions.

Pearson’s (r) correlation analyses were performed between the evaluated variables in the NISS and ISS groups. The correlation method was selected according to whether the data were normally distributed.

For continuous data analysis, multivariate permutation analysis of variance (ANOVA) was used with the lm.rrpp function from the RRPP (Randomized Residual Permutation Procedure) package in R (version 4.5.3) [41]. This method is non-parametric; instead of comparing the observed F-statistics to a theoretical distribution, it empirically constructs the null distribution through permutations of the reduced model residuals. Three independent causal models were specified, each designed to answer a different biological question. In all cases, the group factor (ISS/NISS) was included as a primary predictor, and group × predictor interactions were tested to determine whether the effect of each biomarker on vascularization differed between the two tumor types. Type I (sequential) sum of squares was used, in which each term is tested given all terms previously entered into the model.

The order of entry in each model respects the biological causal chain, so that the regulators most distal to the response variable are introduced first. This design allows the effect of each term to be interpreted as the variance it explains, independent of the preceding causal steps. Parameters for all models: number of permutations: 9999, sum of squares: Type I (sequential), estimation: ordinary least squares (OLSs), reported effect size: partial R^2^ and Z-score (see Supplementary Material S1 for complete ANOVA tables).

Model A assesses whether the relationship between MMP and VEGF-A signaling and its receptor VEGFR-2 alters the angiogenic response (TNVs) according to sarcoma type. Model B does not include VEGF-A; therefore, any significant effect on TNVs is independent of the VEGF-A/VEGFR-2 pathway. Thus, it would be attributable to the PDGFR-α or FGFR-2 pathway. In model C, unlike models A and B, the response variable is not TNVs but the co-expression of MMP-2 and MMP-9, because that is the direct biological relationship regulated by TIMP. A bivariate response is used that preserves the covariance between both MMP, since they share substrate.

## 3. Results

### 3.1. Percentage of Identity of the Proteins of Interest

The percentage of identity between the proteins of interest in *F. catus* and the species against which the antibodies were originally generated was assessed using the BLAST^®^ tool (Basic Local Alignment Search Tool, National Center for Biotechnology Information; https://www.ncbi.nlm.nih.gov/protein/, accessed on 20 June 2026). The results of the sequence analyses for each primary antibody are summarized below.

The anti-MMP-2 antibody was generated against a synthetic peptide derived from human MMP-2. Protein sequence alignment between human MMP-2 (NP_004521.1) and domestic cat (*F. catus*) MMP-2 (XP_003998091.2) showed 97.27% amino acid identity, 100% sequence coverage, and an E-value of 0, indicating a high degree of sequence conservation between species (Supplementary Material S2).

Similarly, alignment of human MMP-9 (NP_004985.2) and *F. catus* MMP-9 (XP_003983461.2) showed an amino acid identity of 82.04%, a sequence coverage of 100%, and an E-value of 0 (Supplementary Material S3).

For TIMP-1, comparison between the human protein (NP_003245.1) and the *F. catus* protein (XP_023105059.1) revealed an amino acid identity of 80.68%, a sequence coverage of 100%, and an E-value of 1 × 10^−131^ (Supplementary Material S4). Furthermore, the alignment of human TIMP-2 (NP_003245.1) and feline TIMP-2 (XP_023105059.1) showed an amino acid identity of 95.48%, a query sequence coverage of 100%, and an E-value of 1 × 10^−164^, indicating a high degree of conservation between species (Supplementary Material S5).

The comparison of human VEGF-A (NP_001303939.1) sequences with feline (*F. catus*) VEGF-A (NP_001422838.1) demonstrated an amino acid identity of 83.18%, a sequence coverage of 100%, and an E-value of 1 × 10^−124^ (Supplementary Material S6). Human VEGFR-2 (NP_002244.1) and feline (*F. catus*) VEGFR-2 (XP_011280181.3) shared 93.29% amino acid identity, 100% sequence coverage, and an E value of 0 (Supplementary Material S7).

Similarly, human FGFR-2 (NP_000132.3) and feline (*F. catus*) FGFR-2 (XP_044896695.1) showed 97.56% amino acid identity, 100% sequence coverage, and an E value of 0 (Supplementary Material S8). Comparison of the human CD31 protein (NP_000433.4) with the feline CD31 protein (*F. catus*) (XP_019673673.2) showed an amino acid identity of 70.77%, a sequence coverage of 100%, and an E-value of 0 (Supplementary Material S9). Finally, alignment of the human PDGFR-α protein (NP_006197.1) and the feline PDGFR-α protein (XP_023108870.1) revealed an amino acid identity of 95.68%, a sequence coverage of 100%, and an E-value of 0 (Supplementary Material S10).

A sequence identity ≥ 70% is generally considered indicative of protein conservation across species. Overall, high to very high sequence similarity was observed between *F. catus* proteins and their corresponding human proteins, supporting antibody cross-reactivity and suitability for immunohistochemical analysis in the present study.

### 3.2. Immunohistochemical Expression

Immunohistochemical expression of all studied variables was confirmed in both ISS and NISS. In 65% of neoplastic cells in both ISS and NISS expressed MMP-2; 65% of ISS and 60% of NISS expressed MMP-9; VEGF-A expression was 95% in ISS neoplastic cells and 90% in NISS, though its receptor VEGF-R2 was only expressed in 40% of ISS neoplastic cells and 75% of NISS tumor cells; PDGFR-α showed a high percentage of immunostaining in the neoplastic cells of both groups (100% ISS, 90% NISS); and FGFR-2 was expressed in 55% of ISSs and 90% of NISSs.

**Figure 1:**
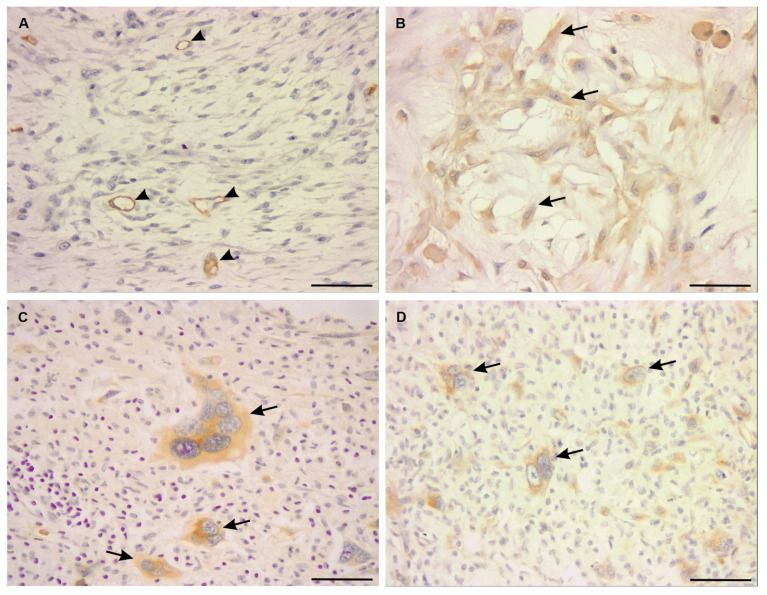
(**A**) Blood vessels labeled with anti-CD31. (**B**) Cytoplasmic labeling with anti-FGFR-2. (**C**) Cytoplasmic labeling with anti-MMP-9. (**D**) Cytoplasmic labeling with anti-TIMP-1. Scale bar: 200 µm (**A**); 50 µm (**B**–**D**).

Table 3 shows the immunohistochemical expression results for the different variables evaluated in both sarcoma groups (ISS and NISS). Immunostaining for FGFR-2, PDGFR-α, VEGF-A, and VEGFR-2 was positive in the cytoplasm of neoplastic and vascular cells. Figure 1 shows the immunostaining for CD31, FGFR-2, MMP-9, and TIMP-1.

### 3.3. Statistical Analysis

Of the qualitative variables evaluated, FGFR-2 showed significant differences (*p* < 0.05; Table 3) where the expression was higher in NISS. VEGFR-2 showed a trend (0.05 < *p* < 0.10) where the expression was also higher in NISS (Table 3). In contrast, MMP-2 and MMP-9 were expressed at almost identical frequencies in both groups, and TIMP-1 and TIMP-2 were somewhat more frequent in ISS without approaching significance. VEGF-A and PDGFR-α were excluded from the comparative analysis because they were almost universally expressed, and limited the power of the test for these markers. Table 4 summarizes the differences in means for quantitative variables. Table 5 summarizes the results of the correlation analyses.

MMP-2 expression showed a positive correlation with VEGF-A and TIMP-1 expression in ISS (r = 0.504 and 0.597, respectively), and with FGFR-2 expression in NISS (r = 0.588). MMP-9 expression was positively correlated with MMP-2 expression in both ISS and NISS (r = 0.503 and 0.665, respectively), as well as with TIMP-1 expression in ISS (r = 0.751). Similarly, TIMP-1 expression was positively correlated with VEGF-A and PDGFR-α expression in ISS (r = 0.568 and 0.451, respectively). In ISS, PDGFR-α expression was negatively correlated with the total number of vessels (TNVs) (r = −0.630).

According to model A, there are significant differences in the TNVs between the two groups (ISS and NISS) (*p* = 0.0448); however, these differences cannot be explained by any of the variables studied (MMP-2, MMP-9, VEGF-A, and VEGFR-2). Model B showed that there are significant differences in the total number of vessels between the two groups (ISS and NISS) (*p* = 0.0381); however, these differences cannot be explained by any of the pathways studied (PDGFR-α, FGFR-2).

Finally, according to model C, the combined expression of MMP-2 and MMP-9 did not differ between the two tumor groups. TIMP-1 expression significantly explains (*p* = 0.0004) the immunostaining pattern of MMP-2 and MMP-9 in both groups combined. Furthermore, the effect of TIMP-1 on MMP expression differed significantly between the groups (*p* = 0.0119); in other words, TIMP-1 regulates MMP expression differently in both groups.

## 4. Discussion

This study demonstrated that both ISSs and NISSs express MMP-2 and MMP-9. Our results, obtained with a larger number of cases, were consistent with those of Wojtkowska et al. [8] and Porcellato et al. [14]. TIMP-2 expression was also demonstrated in ISSs, as described by Porcellato et al. [14]. In our study, NISSs also expressed TIMP-2. TIMP-1 was found in both ISSs and NISSs. No studies in the available literature have examined TIMP-1 expression in feline sarcomas or TIMP-2 expression in NISS.

Both tumor groups expressed PDGFR-α and FGFR-2, which is consistent with the findings of previous work by the group [26]. Both ISSs and NISSs expressed VEGF-A and its receptor VEGFR-2. To date, we have not found any publications that have evaluated the role of the VEGF pathway in feline sarcomas.

FGFR-2 immunolabeling was the only variable that showed significant differences in all applied statistical methods, even after adjusting *p*-values using the Benjamini–Hochberg false discovery rate (FDR) procedure. This receptor was expressed at higher levels in NISSs than in ISSs. Further studies are needed to evaluate the effects of FGFR-2 activation in neoplastic cells. In ISSs, a positive correlation between FGFR-2 expression and cell proliferation has previously been demonstrated [26].

A positive correlation was detected between MMP-2 expression and the expression of VEGF-A and TIMP-1 in ISSs, as well as between MMP-2 expression and FGFR-2 in NISS. The positive correlations observed between MMP-2 and VEGF-A in ISS and between MMP-2 and FGFR-2 in NISS could be explained by one of the biological functions of MMP-2: the degradation of the extracellular matrix (ECM), which acts as a reservoir of biologically active molecules. This process promotes the release of angiogenic growth factors, including VEGF-A and FGF-related signaling molecules, thereby facilitating tumor progression and angiogenesis [17,42].

MMP-9 expression was positively correlated with MMP-2 expression in both ISS and NISS. In the case of NISS, these findings are consistent with those reported by other authors [8,21], whereas different results have been described for ISS. In ISS, this discrepancy may be attributable to the smaller sample size used in previous studies compared with that of the present work. Co-expression of these enzymes has been reported in several human tumors, including sarcomas [15,43,44]. Furthermore, MMP-2 is capable of activating pro-MMP-9 into its active form, MMP-9, which may explain the positive correlation observed between the expression levels of these two enzymes [42].

MMP-9 expression was positively correlated with TIMP-1 expression in ISS. TIMP-1 acts as an inhibitor of both MMP-9 and MMP-2 [13,19]; therefore, its expression may increase in response to the overexpression of these metalloproteinases. This relationship may explain the positive correlation observed between these variables in ISS. Furthermore, in Model C, which evaluated MMP-2 and MMP-9 expression, TIMP-1 expression significantly contributed to explaining the immunolabeling patterns of both MMP across the tumor groups. Since the effect of TIMP-1 on these metalloproteinases differed significantly between groups, this difference may account for the fact that correlations between MMP and TIMP-1 were observed only in ISS.

TIMP-1 expression was positively correlated with VEGF-A and PDGFR-α expression in ISS. TIMP-1 has been reported to promote the expression of VEGF-A [43], which may explain the positive association observed between these two molecules. In addition, TIMP-1 expression is known to increase during inflammatory processes [18]. A study conducted in mice demonstrated that PDGFR-α-positive cells were the primary source of TIMP-1 in pulmonary tissue following infection with influenza A virus [45]. A similar relationship may occur in the neoplastic cells of ISS, potentially explaining the positive correlation observed between PDGFR-α and TIMP-1 expression.

PDGFR-α expression in ISS was negatively correlated with TNV. Given the proliferative effects associated with PDGFR-α activation and its overexpression in neoplastic cells, it is plausible that neoplastic cell proliferation predominates over vascular proliferation. Consequently, the area occupied by neoplastic cells increases relative to the vascular area, resulting in a lower number of vessels per unit area evaluated. A similar finding has previously been reported in ISS [35].

Models A and B demonstrated that none of the pathways evaluated were able to explain the differences in TNVs between the two tumor groups. This finding may indicate that angiogenesis in both sarcoma types is driven by alternative pathways, such as those mediated by hypoxia-inducible factor (HIF), transforming growth factor-beta (TGF-β), or interleukin-8 (IL-8). The HIF-mediated pathway is particularly active in certain neoplasms, especially solid tumors, where the accumulation of HIF-1 promotes the expression of angiogenesis-related genes [25]. In addition, the release of TGF-β through the action of metalloproteinases, particularly MMP-2 and MMP-9, has been shown to induce angiogenesis [25]. Furthermore, the secretion of specific interleukins, such as IL-8, by both neoplastic and inflammatory cells associated with the tumor microenvironment, contributes to angiogenesis. Increased IL-8 expression has been associated with a strong pro-angiogenic effect [46].

It would have been of interest to correlate the obtained results with relevant clinical outcome data, such as survival time, recurrence rates, and the presence of metastasis. However, it was not possible to access this information. In the cases where some clinical data were available, the records were incomplete and limited to only a small number of the cases included in the study. Consequently, the available information lacked sufficient validity for meaningful analysis and was therefore not included in the present study.

## 5. Conclusions

The ability to induce blood vessel formation is essential for neoplastic progression. Inhibition of angiogenesis has become an important therapeutic strategy for the treatment of various neoplasms and may also be beneficial in soft tissue sarcomas of domestic cats. Continued research into the pathways regulating angiogenesis in these tumors is crucial for identifying potential therapeutic targets and developing strategies to modulate the angiogenic response, thereby limiting tumor progression.

According to our results, FGFR-2 appears to be a molecule of interest for future studies aimed at elucidating its role in feline sarcomas and evaluating its potential as a therapeutic target. Given that FGFR-2 has been shown to stimulate neoplastic cell proliferation in ISS, a similar mitogenic effect may also occur in NISS. If confirmed, this receptor could represent a potential therapeutic target for inhibiting tumor cell proliferation in this group of sarcomas. Further studies are needed to investigate the relationship between FGFR-2 expression and cell proliferation in NISS. Selective FGFR-2 inhibitors are currently being evaluated for their antineoplastic effects in human solid tumors [47,48]. To the best of our knowledge, no studies have evaluated FGFR-2 inhibitors in animals. However, because the finding of the present study are based on immunohistochemical analysis of a small number of cases, studies involving larger cohorts, as well as in vitro experiments using feline sarcoma cell lines, are needed to confirm the functional relevance of this receptor.

NISS are poorly characterized neoplasms, and consequently, therapeutic options for their management remain limited. Although surgical excision is currently considered the treatment of choice, these tumors are locally invasive, and affected patients may benefit from adjuvant therapies aimed at preventing recurrence or prolonging the tumor-free interval. In this context, the findings of the present study may provide a basis for future research into the pathogenesis of these tumors and the development of novel therapeutic approaches for NISS.

## Figures and Tables

**Table 1:** Anatomical location of sarcomas.

NISS (%)		ISS (%)	
Auricular pinna	4 (20%)	Costal region (thoracic wall)	13 (65%)
Abdomen	2 (10%)	Flank	3 (15%)
Plantar pad	1 (5%)	Lumbar region	1 (5%)
Ear base	1 (5%)	Lumbosacral region	1 (5%)
Arm	1 (5%)	Dorsal neck region	1 (5%)
Elbow	1 (5%)	Thorax	1 (5%)
Dorsum of the hand	1 (5%)		
Sternal	1 (5%)		
Inguinal region	1 (5%)		
Lip	1 (5%)		
Digital mass	1 (5%)		
Perineum	1 (5%)		
Carpus	2 (10%)		
Head	2 (10%)		

**Table 2:** Primary and secondary antibodies: dilutions and specifications.

Primary Antibody			Secondary Antibody
Name	Specifications	Dilution	Biotinylated dual anti-rabbit/anti-mouse (BioGenex, HK340. San Ramón, CA, USA); ready to use)
MMP-2	Rabbit Polyclonal Abcam, ab97779	1:400	
MMP-9	Rabbit Polyclonal, Abcam, ab74277	1:400	
TIMP-1	Rabbit Polyclonal, sc-6832R	1:400	
TIMP-2	Mouse Monoclonal, Abcam, ab1828	1:200	
VEGF-A	Mouse Monoclonal, Clone VG-1	1:100	
VEGFR-2	Rabbit Monoclonal, Clone 55B11	1:200	
FGF-R2	Rabbit Polyclonal Abcam, ab126861	1:100	
CD-31	Rabbit Polyclonal, Abcam, ab28364	1:25	
PDGFR-α	Rabbit Polyclonal, MBS301499	1:100	

**Table 3:** Frequency and proportion of positive (Pos) and negative (Neg) expression by group (*N* = 20 per group).

*p*	OR (95% CI)	NISS		ISS		Variable
		Neg	Pos	Neg	Pos	
1.0000	1.00 (0.23–4.45)	7 (35%)	13 (65%)	7 (35%)	13 (65%)	MMP-2
1.0000	0.81 (0.19–3.49)	8 (40%)	12 (60%)	7 (35%)	13 (65%)	MMP-9
0.3332	0.44 (0.10–1.86)	10 (50%)	10 (50%)	6 (30%)	14 (70%)	TIMP-1
0.2049	0.37 (0.08–1.53)	12 (60%)	8 (40%)	7 (35%)	13 (65%)	TIMP-2
0.0536	4.32 (0.98–21.95)	5 (25%)	15 (75%)	12 (60%)	8 (40%)	VEGFR-2
0.0138	8.49 (1.41–94.80)	2 (10%)	18 (90%)	10 (50%)	10 (50%)	FGFR-2
—	—	2 (10%)	18 (90%)	1 (5%)	19 (95%)	VEGF-A
—	—	3 (15%)	17 (85%)	0 (0%)	20 (100%)	PDGFR-α

OR = odds ratio; (95% CI) = 95% confidence interval.

**Table 4:** Differences in means for quantitative variables.

Variable	Unadjusted *p* Values. t from Welch Mann–Whitney U.	Adjusted *p*-Values Benjamini–Hochberg, FDR
MMP-2	ISS > NISS t: 0.1447 MW: 0.3029	t: 0.8942 MW: 0.7610
MMP-9	ISS > NISS t: 0.0402 * MW: 0.2061	t: 0.9447 MW: 0.8058
TIMP-1	ISS > NISS t: 0.0110 * MW: 0.0348 *	t: 0.0440 * MW: 0.0964
VEGFR-2	ISS < NISS t: 0.9136 MW: 0.2103	t: 0.9447 MW: 0.3029
FGFR-2	ISS < NISS t: 0.0001 *** MW: 0.0002 ***	t: 0.0004 *** MW: 0.0020 **
TNVs	ISS < NISS t: 0.0223 * MW: 0.0336 *	t: 0.0508 MW: 0.0812

* (*p* < 0.05) ** (*p* < 0.01). *** (*p* < 0.001).

**Table 5:** Correlation analysis.

NISS	ISS	Variable
Correlation (+) whit FGFR-2 (r = 0.588)	Correlation (+) whit VEGF-A and TIMP-1 (r = 0.504; r = 0.597)	MMP-2
Correlation (+) whit FGFR-2 (r = 0.665)	Correlation (+) whit MMP-2 and TIMP-1 (r = 0.503; r = 0.751)	MMP-9
NC	Correlation (+) with VEGF-A and PDGFR-α (r = 0.568; r = 0.451)	TIMP-1
NC	Correlation (−) with TNVs (r = −0.630)	PDGFR-α

NC: No correlation; Pearson correlation coefficients among biomarkers (only significant correlations are shown).

## Data Availability

The original contributions presented in this study are included in the article. Further inquiries can be directed to the corresponding authors.

## Supplementary Materials

The following supporting information can be downloaded at: https://www.mdpi.com/article/10.3390/biology15151322/s1, Supplementary Material S1: Multivariate permutation ANOVA (RRPP) for the three models. Terms are listed in their order of entry; Type I (sequential) sums of squares, 9999 permutations. Biomarkers variables were square-root transformed and TNV was log-transformed; Supplementary Material S2: Protein BLAST alignment between human MMP-2 (NP_004521.1) and feline MMP-2 (XP_003998091.2), showing 97.27% amino acid identity, 100% query coverage and an E-value of 0; Supplementary Material S3: Protein BLAST alignment between human MMP-9 (NP_004985.2) and feline MMP-9 (XP_003983461.2), showing 82.04% amino acid identity, 100% query coverage and an E-value of 0; Supplementary Material S4: Protein BLAST alignment between human TIMP-1 (NP_003245.1) and feline TIMP-1 (XP_023105059.1), showing 80.68% amino acid identity, 100% query coverage and an E-value of 1 × 10^−131^; Supplementary Material S5: Protein BLAST alignment between human TIMP-2 (NP_003245.1) and feline TIMP-2 (XP_023105059.1), showing 95.48% amino acid identity, 100% query coverage and an E-value of 1 × 10^−164^; Supplementary Material S6: Protein BLAST alignment between human VEGF-A (NP_001303939.1) and feline VEGF-A (NP_001422838.1), showing 83.18% amino acid identity, 100% query coverage and an E-value of 1 × 10^−124^; Supplementary Material S7: Protein BLAST alignment between human VEGFR-2 (NP_002244.1) and feline VEGFR-2 (XP_011280181.3), showing 93.29% amino acid identity, 100% query coverage and an E-value of 0; Supplementary Material S8: Protein BLAST alignment between human FGFR-2 (NP_000132.3) and feline FGFR-2 (XP_044896695.1), showing 97.56% amino acid identity, 100% query coverage and an E-value of 0; Supplementary Material S9: Protein BLAST alignment between human CD-31 (NP_000433.4) and feline CD-31 (XP_019673673.2), showing 70.77% amino acid identity, 100% query coverage and an E-value of 0; Supplementary Material S10: Protein BLAST alignment between human PDGFR-α (NP_006197.1) and feline PDGFR-α (XP_023108870.1), showing 95.68% amino acid identity, 100% query coverage and an E-value of 0.
