# Supplementary material for: Microenvironment of Sarcomas Associated and Not Associated with Inoculation Sites in Domestic Cats: The Role of Metalloproteinases and Angiogenic Factors
Source: Biology (Basel). 2026 Aug 6;15(15):1322. doi: 10.3390/biology15151322 (PMC13464654; doi:10.3390/biology15151322)
Supplement: Supplementary file 1 [file biology-15-01322-s001.zip › Supplementary Material S1.pdf]

Supplementary Material S1. Multivariate permutation ANOVA (RRPP) for the three models. Terms are listed in their order of entry; Type I (sequential) sums of squares, 9999 permutations. Biomarkers variables were square-root transformed and TNV was log-transformed.

**Model A — response: log(TNV)**							
**Term**	**df**	**SS**	**MS**	**R²**	***F***	***Z***	***p***
Group	1	1.207	1.2070	0.103	4.546	1.640	0.0448 *
√MMP-2	1	0.202	0.2018	0.017	0.760	0.354	0.3749
√MMP-9	1	0.318	0.3178	0.027	1.197	0.621	0.2841
√VEGF-A	1	0.252	0.2517	0.022	0.948	0.484	0.3270
√VEGFR-2	1	0.037	0.0372	0.003	0.140	−0.571	0.7036
Group × √MMP-2	1	0.729	0.7294	0.062	2.747	1.232	0.1094
Group × √MMP-9	1	0.408	0.4075	0.035	1.535	0.798	0.2243
Group × √VEGF-A	1	0.004	0.0040	0.000	0.015	−1.359	0.8977
Group × √VEGFR-2	1	0.575	0.5746	0.049	2.164	1.025	0.1583
Residuals	30	7.965	0.2655	0.681			
Total	39	11.696					
**Model B — response: log(TNV)**							
**Term**	**df**	**SS**	**MS**	**R²**	***F***	***Z***	***p***
Group	1	1.207	1.2070	0.103	4.807	1.701	0.0381 *
√PDGFR-α	1	0.561	0.5609	0.048	2.234	1.096	0.1414
√FGFR-2	1	0.102	0.1023	0.009	0.408	−0.047	0.5307
Group × √PDGFR-α	1	0.863	0.8635	0.074	3.439	1.425	0.0754
Group × √FGFR-2	1	0.425	0.4245	0.036	1.691	0.858	0.2082
Residuals	34	8.538	0.2511	0.730			
Total	39	11.696					
**Model C — response: (√MMP-9, √MMP-2)**							
**Term**	**df**	**SS**	**MS**	**R²**	***F***	***Z***	***p***
Group	1	0.235	0.2347	0.044	2.370	1.223	0.1092
√TIMP-1	1	1.126	1.1262	0.211	11.375	3.169	0.0004 ***
√TIMP-2	1	0.014	0.0136	0.003	0.137	−1.097	0.8614
Group × √TIMP-1	1	0.510	0.5101	0.096	5.152	2.219	0.0119 *
Group × √TIMP-2	1	0.085	0.0849	0.016	0.858	0.234	0.4097
Residuals	34	3.366	0.0990	0.631			
Total	39	5.336					

* $p < 0.05$; *** $p < 0.001$. R^2 is the partial coefficient of determination for each term. Z is the standardised effect size derived from the permutation distribution.
