# Supplementary material for: Microenvironment of Sarcomas Associated and Not Associated with Inoculation Sites in Domestic Cats: The Role of Metalloproteinases and Angiogenic Factors
Source: Biology (Basel). 2026 Aug 6;15(15):1322. doi: 10.3390/biology15151322 (PMC13464654; doi:10.3390/biology15151322)
Supplement: Supplementary file 1 [file biology-15-01322-s001.zip › Supplementary Material S10.pdf]

Supplementary Material S10. Protein BLAST alignment between human PDGFR-α (NP_006197.1) and feline PDGFR-α (XP_023108870.1), showing 95.68% amino acid identity, 100% query coverage and an E-value of 0.

Score	Expect	Method	Identities	Positives	Gaps
2163 bits(5604)	0.0	Compositional matrix adjust.	1042/1089(96%)	1068/1089(98%)	0/1089(0%

```
Query  1      MGTSHPAFLVLGCLLTGLSLILCQLSLPSILPNENEKVVQLNSSFSLRCFGESEVSWQYP  60
              MGTSH A LVL CLLTG SLI CQLSLPSILPNENEKVVQLNSSFSLRCFGESEVSWQYP
Sbjct  1      MGTSHLALLVLVCLLTGPSLISCQLSLPSILPNENEKVVQLNSSFSLRCFGESEVSWQYP  60

Query  61     MSEEESSDVEIRNEENNSGLFVTVLEVSSASAAHTGLYTCYYNHTQTEENELEGRHIYIY  120
              MSEEE+ +VE+RNEENNSGLFVTVLEV +ASAAHTGLYTCYYNHTQT+ENE+EGR IYIY
Sbjct  61     MSEEENPNVEVRNEENNSGLFVTVLEVVNASAAHTGLYTCYYNHTQTDENEIEGRRIYIY  120

Query  121    VPDPDVAFVPLGMTDYLVIVEDDDSAIIPCRTTDPETPVTLHNSEGVVPASYDSRQGFNG  180
              VPDPDVAFVPLGMTDYLVIVEDDDSAIIPCRTTDP+TPVTL +S+GVV ASYDSRQGFNG
Sbjct  121    VPDPDVAFVPLGMTDYLVIVEDDDSAIIPCRTTDPKTPVTLRSSDGVVHASYDSRQGFNG  180

Query  181    TFTVGPYICEATVKGKKFQTIPFNVYALKATSELDLEMEALKTVYKSGETIVVTCAVFNN  240
              TF+VGPYICEATV GKKFQTIPFNVYALKATSELDLEMEALKTVYKSGETIVVTCAVFNN
Sbjct  181    TFSVGPYICEATVSGKKFQTIPFNVYALKATSELDLEMEALKTVYKSGETIVVTCAVFNN  240

Query  241    EVVDLQWTYPGEVKGKGITMLEEIKVPSIKLVYTLTVPEATVKDSGDYECAARQATREVK  300
              EVVDLQWTYPGEVKGKGITMLEEIKVPSIKLVYTLTVPEATVKDSGDYECAARQAT+EVK
Sbjct  241    EVVDLQWTYPGEVKGKGITMLEEIKVPSIKLVYTLTVPEATVKDSGDYECAARQATKEVK  300

Query  301    EMKKVTISVHEKGFIEIKPTFSQLEAVNLHEVKHFVVEVRAYPPPRISWLKNNLTLIENL  360
              EMKKVTISVHEKGFIEIKP FS LEAV+LHEVKHFVV+V+AYPPPRISWLK+NLTLIENL
Sbjct  301    EMKKVTISVHEKGFIEIKPNFSPLEAVDLHEVKHFVVDVQAYPPPRISWLKDNLTLIENL  360

Query  361    TEITTDVEKIQEIRYRSKLKLIRAKEEDSGHYTIVAQNEDAVKSYTFELLTQVPSSILDL  420
              TEITTD+EKIQE+RYRSKLKLIRAKEEDSGHYTIV QNED VKSYTFELLTQVPSSILDL
Sbjct  361    TEITTDIEKIQEVRYRSKLKLIRAKEEDSGHYTIVVQNEDDVKSYTFELLTQVPSSILDL  420

Query  421    VDDHHGSTGGQTVRCTAEGTPLPDIEWMICKDIKKCNNETSWTILANNVSNIITEIHSRD  480
              VDDHHGSTGGQTVRCTAEGTPLPDIEWMICKDIKKCNNETSWT+LANN+SNIITE+H RD
Sbjct  421    VDDHHGSTGGQTVRCTAEGTPLPDIEWMICKDIKKCNNETSWTVLANNISNIITEVHRRD  480

Query  481    RSTVEGRVTFAKVEETIAVRCLAKNLLGAENRELKLVAPTLRSELTVAAAVLVLLVIVII  540
              RSTVEGRVTF +VEETIAVRCLAKNLLGAENRELKLVAPTLRSELTVAAAVLVLLVIVII
Sbjct  481    RSTVEGRVTFTRVEETIAVRCLAKNLLGAENRELKLVAPTLRSELTVAAAVLVLLVIVII  540

Query  541    SLIVLVVIWKQKPRYEIRWRVIESISPDGHEYIYVDPMQLPYDSRWEFPRDGLVLGRVLG  600
              SLIVLVVIWKQKPRYEIRWRVIESISPDGHEYIYVDPMQLPYDSRWEFPRDGLVLGR+LG
Sbjct  541    SLIVLVVIWKQKPRYEIRWRVIESISPDGHEYIYVDPMQLPYDSRWEFPRDGLVLGRILG  600

Query  601    SGAFGKVVEGTAYGLSRSQPVMKVAVKMLKPTARSSEKQALMSELKIMTHLGPHLNIVNL  660
              SGAFGKVVEGTAYGLSRSQPVMKVAVKMLKPTARSSEKQALMSELKIMTHLGPHLNIVNL
Sbjct  601    SGAFGKVVEGTAYGLSRSQPVMKVAVKMLKPTARSSEKQALMSELKIMTHLGPHLNIVNL  660

Query  661    LGACTKSGPIYIITEYCFYGDLVNYLHKNRDSFLSHHPEKPKKELDIFGLNPADESTRSY  720
              LGACTKSGPIYIITEYCFYGDLVNYLHKNRDSFLS HPEKPKKELDIFGLNPADESTRSY
Sbjct  661    LGACTKSGPIYIITEYCFYGDLVNYLHKNRDSFLSRHPEKPKKELDIFGLNPADESTRSY  720

Query  721    VILSFENNGDYMDMKQADTTQYVPMLERKEVSKYSDIQRSLYDRPASYKKKSMLDSEVKN  780
              VILSFENNGDYMDMKQADTTQYVPMLERKEVSKYSDIQRSLYDRPASYKKKS  DSEVKN
Sbjct  721    VILSFENNGDYMDMKQADTTQYVPMLERKEVSKYSDIQRSLYDRPASYKKKSTSDSEVKN  780

Query  781    LLSDDNSEGLTLLDLLSFTYQVARGMEFLASKNCVHRDLAARNVLLAQGKIVKICDFGLA  840
              LLSDDNSEGLTLLDLLSFTYQVARGMEFLASKNCVHRDLAARNVLLAQGKIVKICDFGLA
Sbjct  781    LLSDDNSEGLTLLDLLSFTYQVARGMEFLASKNCVHRDLAARNVLLAQGKIVKICDFGLA  840

Query  841    RDIMHDSNYVSKGSTFLPVKWMAPESIFDNLYTTLSDVWSYGILLWEIFSLGGTPYPGMM  900
              RDIMHDSNYVSKGSTFLPVKWMAPESIFDNLYTTLSDVWSYGILLWEIFSLGGTPYPGMM
Sbjct  841    RDIMHDSNYVSKGSTFLPVKWMAPESIFDNLYTTLSDVWSYGILLWEIFSLGGTPYPGMM  900

Query  901    VDSTFYNKIKSGYRMAKPDHATSEVYEIMVKCWNSEPEKRPSFYHLSEIVENLLPGQYKK  960
              VDSTFYNKIK+GYRMAKPDHATSEVYEIMVKCW+SEPEKRPSFYHLSEIVENLLPGQYKK
Sbjct  901    VDSTFYNKIKNGYRMAKPDHATSEVYEIMVKCWHSEPEKRPSFYHLSEIVENLLPGQYKK  960

Query  961    SYEKIHLDFLKSDHPAVARMRVDSDNAYIGVTYKNEEDKLKDWEGGLDEQRLSADSGYII  1020
              SYEKIHLDFLKSDHPAVARMRVDSDNAYIGVTYK+EEDKLKDWEG LDEQRLSADSGYII
Sbjct  961    SYEKIHLDFLKSDHPAVARMRVDSDNAYIGVTYKHEEDKLKDWEGSLDEQRLSADSGYII  1020

Query  1021   PLPDIDPVPEEEDLGKRNRHSSQTSEESAIETGSSSSTFIKREDETIEDIDMMDDIGIDS  1080
              PLPDIDPVPEEEDLGKRNRHSSQTSEESAIETGSSSSTF+KREDETIEDIDMMDDIGIDS
Sbjct  1021   PLPDIDPVPEEEDLGKRNRHSSQTSEESAIETGSSSSTFVKREDETIEDIDMMDDIGIDS  1080

Query  1081   SDLVEDSFL  1089
              SDLVEDSFL
Sbjct  1081   SDLVEDSFL  1089
```
