# Supplementary material for: Microenvironment of Sarcomas Associated and Not Associated with Inoculation Sites in Domestic Cats: The Role of Metalloproteinases and Angiogenic Factors
Source: Biology (Basel). 2026 Aug 6;15(15):1322. doi: 10.3390/biology15151322 (PMC13464654; doi:10.3390/biology15151322)
Supplement: Supplementary file 1 [file biology-15-01322-s001.zip › Supplementary Material S2.pdf]

Supplementary Material S2. Protein BLAST alignment between human MMP-2 (NP_004521.1) and feline MMP-2 (XP_003998091.2), showing 97.27% amino acid identity, 100% query coverage and an E-value of 0.

Score	Expect	Method	Identities	Positives	Gaps
1333 bits(3449)	0.0	Compositional matrix adjust.	641/659(97%)	649/659(98%)	0/659(0%

```
Query  2    EALMARGALTGPLRALCLLGCLLSHAAAAPSPIIKFPGDVAPKTDKELAVQYLNTFYGCP  61
            EA  ARGAL GPLRALC+LGCLL  AAAAPSPIIKFPGDVAPKTDKELAVQYLNTFYGCP
Sbjct  3    EARGARGALAGPLRALCVLGCLLGRAAAAPSPIIKFPGDVAPKTDKELAVQYLNTFYGCP  62

Query  62   KESCNLFVLKDTLKKMQKFFGLPQTGDLDQNTIETMRKPRCGNPDVANYNFFPRKPKWDK  121
            KESCNLFVLKDTLKKMQKFFGLPQTGDLDQ+TIETMRKPRCGNPDVANYNFFPRKPKWDK
Sbjct  63   KESCNLFVLKDTLKKMQKFFGLPQTGDLDQSTIETMRKPRCGNPDVANYNFFPRKPKWDK  122

Query  122  NQITYRIIGYTPDLDPETVDDAFARAFQVWSDVTPLRFSRIHDGEADIMINFGRWEHGDG  181
            NQITYRIIGYTPDLDPETVDDAFARAFQVWSDVTPLRFSRIHDGEADIMINFGRWEHGDG
Sbjct  123  NQITYRIIGYTPDLDPETVDDAFARAFQVWSDVTPLRFSRIHDGEADIMINFGRWEHGDG  182

Query  182  YPFDGKDGLLAHAFAPGTGVGGDSHFDDDELWTLGEGQVVRVKYGNADGEYCKFPFLFNG  241
            YPFDGKDGLLAHAFAPG GVGGDSHFDDDELWTLGEGQVVRVKYGNADGEYCKFPFLFNG
Sbjct  183  YPFDGKDGLLAHAFAPGPGVGGDSHFDDDELWTLGEGQVVRVKYGNADGEYCKFPFLFNG  242

Query  242  KEYNSCTDTGRSDGFLWCSTTYNFEKDGKYGFCPHEALFTMGGNAEGQPCKFPFRFQGTS  301
            KEY SCTDTGRSDGFLWCSTTYNFEKDGKYGFCPHEALFTMGGNA+GQPCKFPFRFQGTS
Sbjct  243  KEYTSCTDTGRSDGFLWCSTTYNFEKDGKYGFCPHEALFTMGGNADGQPCKFPFRFQGTS  302

Query  302  YDSCTTEGRTDGYRWCGTTEDYDRDKKYGFCPETAMSTVGGNSEGAPCVFPFTFLGNKYE  361
            YDSCTTEGRTDGYRWCGTTEDYDRDKKYGFCPETAMSTVGGNSEGAPCVFPFTFLG+K+E
Sbjct  303  YDSCTTEGRTDGYRWCGTTEDYDRDKKYGFCPETAMSTVGGNSEGAPCVFPFTFLGSKHE  362

Query  362  SCTSAGRSDGKMWCATTANYDDDRKWGFCPDQGYSLFLVAAHEFGHAMGLEHSQDPGALM  421
            SCTSAGRSDGKMWCATTANYDDDRKWGFCPDQGYSLFLVAAHEFGHAMGLEHSQDPGALM
Sbjct  363  SCTSAGRSDGKMWCATTANYDDDRKWGFCPDQGYSLFLVAAHEFGHAMGLEHSQDPGALM  422

Query  422  APIYTYTKNFRLSQDDIKGIQELYGASPDIDLGTGPTPTLGPVTPEICKQDIVFDGIAQI  481
            APIYTYTKNFRLS DDIKGIQELYGASPDID GTGPTPTLGPVTPEICKQDIVFDGI+QI
Sbjct  423  APIYTYTKNFRLSHDDIKGIQELYGASPDIDTGTGPTPTLGPVTPEICKQDIVFDGISQI  482

Query  482  RGEIFFFKDRFIWRTVTPRDKPMGPLLVATFWPELPEKIDAVYEAPQEEKAVFFAGNEYW  541
            RGEIFFFKDRFIWRTVTPR KPMGPLLVATFWPELPEKIDAVYEAPQEEKAVFFAGNEYW
Sbjct  483  RGEIFFFKDRFIWRTVTPRSKPMGPLLVATFWPELPEKIDAVYEAPQEEKAVFFAGNEYW  542

Query  542  IYSASTLERGYPKPLTSLGLPPDVQRVDAAFNWSKNKKTYIFAGDKFWRYNEVKKKMDPG  601
            +YSASTLERGYPKPLTSLGLPPDVQRVDAAFNWSKN+KTYIFAGDKFWRYNEVKKKMDPG
Sbjct  543  VYSASTLERGYPKPLTSLGLPPDVQRVDAAFNWSKNRKTYIFAGDKFWRYNEVKKKMDPG  602

Query  602  FPKLIADAWNAIPDNLDAVVDLQGGGHSYFFKGAYYLKLENQSLKSVKFGSIKSDWLGC  660
            FPKLIADAWNAIPDNLDAVVDLQGGGHSYFFKGAYYLKLENQSLKSVKFGSIKSDWLGC
Sbjct  603  FPKLIADAWNAIPDNLDAVVDLQGGGHSYFFKGAYYLKLENQSLKSVKFGSIKSDWLGC  661
```
