# Supplementary material for: Microenvironment of Sarcomas Associated and Not Associated with Inoculation Sites in Domestic Cats: The Role of Metalloproteinases and Angiogenic Factors
Source: Biology (Basel). 2026 Aug 6;15(15):1322. doi: 10.3390/biology15151322 (PMC13464654; doi:10.3390/biology15151322)
Supplement: Supplementary file 1 [file biology-15-01322-s001.zip › Supplementary Material S3.pdf]

Supplementary Material S3. Protein BLAST alignment between human MMP-9 (NP_004985.2) and feline MMP-9 (XP_003983461.2), showing 82.04% amino acid identity, 100% query coverage and an E-value of 0.

Score	Expect	Method	Identities	Positives	Gaps
1113 bits(2880)	0.0	Compositional matrix adjust.	580/707(82%)	630/707(89%)	2/707(0%)

```
Query  1    MSLWQPLVLVLLVLGCCFAAPRQRQSTLVLFPGDLRTNLTDRQLAEEYLYRYGYTRVAEM  60
            MS  QPLVL LLVLGCC AAPR R+ T+V+FPGDLRTNLTDRQLAEEYLYRYGYT VAE+
Sbjct  1    MSPRQPLVLALLVLGCCTAAPRPRKPTVVVFPGDLRTNLTDRQLAEEYLYRYGYTHVAEI  60

Query  61   RGESKSLGPALLLLQKQLSLPETGELDSATLKAMRTPRCGVPDLGRFQTFEGDLKWHHHN  120
              + +SL   L LLQ++L+LPETGELD  TL+A+RTPRCGVPDLG+FQTFEGDLKWHHHN
Sbjct  61   SDDQQSLSRGLRLLQRRLALPETGELDRTTLEAIRTPRCGVPDLGKFQTFEGDLKWHHHN  120

Query  121  ITYWIQNYSEDLPRAVIDDAFARAFALWSAVTPLTFTRVYSRDADIVIQFGVAEHGDGYP  180
            ITYWIQNYSEDLPR VIDDAFARAFA WSAVTPLTFTRVY  +ADIVIQFGV EHGDGYP
Sbjct  121  ITYWIQNYSEDLPRDVIDDAFARAFAAWSAVTPLTFTRVYGLEADIVIQFGVREHGDGYP  180

Query  181  FDGKDGLLAHAFPPGPGIQGDAHFDDDELWSLGKGVVVPTRFGNADGAACHFPFIFEGRS  240
            FDGKDGLLAHAFPPGPGIQGDAHFDD+ELWSLGKGVVVPT FGNA GAACHFPF FEGRS
Sbjct  181  FDGKDGLLAHAFPPGPGIQGDAHFDDEELWSLGKGVVVPTYFGNAGGAACHFPFTFEGRS  240

Query  241  YSACTTDGRSDGLPWCSTTANYDTDDRFGFCPSERLYTQDGNADGKPCQFPFIFQGQSYS  300
            YSACTTDGRSD + WCSTTA+YDTD RFGFCPSERLYTQDGN +GKPC FPF F+G+SYS
Sbjct  241  YSACTTDGRSDDMLWCSTTADYDTDRRFGFCPSERLYTQDGNGEGKPCVFPFTFEGRSYS  300

Query  301  ACTTDGRSDGYRWCATTANYDRDKLFGFCPTRADSTVMGGNSAGELCVFPFTFLGKEYST  360
            ACTT+GRSDGYRWCATTANYD+DKL+GFCPTRADSTV GGNSAGELCVFPF FLGKEYST
Sbjct  301  ACTTEGRSDGYRWCATTANYDQDKLYGFCPTRADSTVTGGNSAGELCVFPFIFLGKEYST  360

Query  361  CTSEGRGDGRLWCATTSNFDSDKKWGFCPDQGYSLFLVAAHEFGHALGLDHSSVPEALMY  420
            CT EGRGDG LWCATTSNFD DKKWGFCPDQGYSLFLVAAHEFGHALGLDHSSVPEALMY
Sbjct  361  CTREGRGDGHLWCATTSNFDRDKKWGFCPDQGYSLFLVAAHEFGHALGLDHSSVPEALMY  420

Query  421  PMYRFTEGPPLHKDDVNGIRHLYGPRPEPEPRPPTTTTPQPTAPPTVCPTGPPTVHPSER  480
            PMY FTEGPPLH+DDV GI+HLYGPRPEPEPR    TT  P + PTVC TGPPT  P ER
Sbjct  421  PMYSFTEGPPLHEDDVKGIQHLYGPRPEPEPR--PPTTTTPESQPTVCTTGPPTTRPPER  478

Query  481  PTAGPTGPPSAGPTGPPTAGPSTATTVPLSPVDDACNVNIFDAIAEIGNQLYLFKDGKYW  540
            PTAGPTGPPSAGPTGPPTAGPS A TV L P +D CNVN+FDA+AEIGN L+ FKDGKYW
Sbjct  479  PTAGPTGPPSAGPTGPPTAGPSEAPTVSLDPAEDVCNVNVFDAVAEIGNLLHFFKDGKYW  538

Query  541  RFSEGRGSRPQGPFLIADKWPALPRKLDSVFEERLSKKLFFFSGRQVWVYTGASVLGPRR  600
            +FSE  G R +GPFLI +KWPALPRKLDS FE+ L+KK FFFSGRQVWVYTG SVLGPRR
Sbjct  539  QFSENPGRRVRGPFLITNKWPALPRKLDSAFEDPLTKKTFFFSGRQVWVYTGTSVLGPRR  598

Query  601  LDKLGLGADVAQVTGALRSGRGKMLLFSGRRLWRFDVKAQMVDPRSASEVDRMFPGVPLD  660
            LDKLGLG +VAQ+TGAL    GK+LLFS +R WRFDVK Q VD +SA +V++MFPGVPL+
Sbjct  599  LDKLGLGPEVAQITGALPRSGGKVLLFSRQRFWRFDVKTQTVDAKSAGQVEQMFPGVPLN  658

Query  661  THDVFQYREKAYFCQDRFYWRVSSRSELNQVDQVGYVTYDILQCPED  707
            THD+FQ++EKAYFCQDRFYWRVSSR+E+NQVDQVGYVT+D+LQCPED
Sbjct  659  THDIFQFQEKAYFCQDRFYWRVSSRNEVNQVDQVGYVTFDLLQCPED  705
```
