# Supplementary material for: Microenvironment of Sarcomas Associated and Not Associated with Inoculation Sites in Domestic Cats: The Role of Metalloproteinases and Angiogenic Factors
Source: Biology (Basel). 2026 Aug 6;15(15):1322. doi: 10.3390/biology15151322 (PMC13464654; doi:10.3390/biology15151322)
Supplement: Supplementary file 1 [file biology-15-01322-s001.zip › Supplementary Material S4.pdf]

Supplementary Material S4. Protein BLAST alignment between human TIMP-1 (NP_003245.1) and feline TIMP-1 (XP_023105059.1), showing 80.68% amino acid identity, 100% query coverage and an E-value of 1e-131.

Score	Expect	Method	Identities	Positives	Gaps
358 bits(918)	1e-131	Compositional matrix adjust.	167/207(81%)	182/207(87%)	0/207(0%)

```
Query  1     MAPFEPLASGILLLLWLIAPSRACTCVPPHPQTAFCNSDLVIRAKFVGTPEVNQTTLYQR  60
             MAP  PLAS ILLLLWL APSRACTC P HPQTAFC+SD +IRAKFVGT EVNQT L QR
Sbjct  51    MAPLAPLASCILLLLWLAAPSRACTCAPLHPQTAFCSSDFIIRAKFVGTAEVNQTALSQR  110

Query  61    YEIKMTKMYKGFQALGDAADIRFVYTPAMESVCGYFHRSHNRSEEFLIAGKLQDGLLHIT  120
             YEIKMTKM+KGF ALGDA+DIRFVYTP  ESVCGYFHRS NRSEEFLIAGKL++G LHI
Sbjct  111   YEIKMTKMFKGFSALGDASDIRFVYTPTAESVCGYFHRSQNRSEEFLIAGKLRNGHLHIN  170

Query  121   TCSFVAPWNSLSLAQRRGFTKTYTVGCEECTVFPCLSIPCKLQSGTHCLWTDQLLQGSEK  180
             TCS+V PWNSLS +QRRGFTKTY  GCEECTVF C SIPCKLQ+ THCLWTDQ L G++K
Sbjct  171   TCSYVVPWNSLSSSQRRGFTKTYAAGCEECTVFSCSSIPCKLQNDTHCLWTDQFLTGTDK  230

Query  181   GFQSRHLACLPREPGLCTWQSLRSQIA  207
             GFQSRHLACLPREPG+CTWQSLR+++A
Sbjct  231   GFQSRHLACLPREPGICTWQSLRTRMA  257
```
