# Supplementary material for: Microenvironment of Sarcomas Associated and Not Associated with Inoculation Sites in Domestic Cats: The Role of Metalloproteinases and Angiogenic Factors
Source: Biology (Basel). 2026 Aug 6;15(15):1322. doi: 10.3390/biology15151322 (PMC13464654; doi:10.3390/biology15151322)
Supplement: Supplementary file 1 [file biology-15-01322-s001.zip › Supplementary Material S5.pdf]

Supplementary Material S5. Protein BLAST alignment between human TIMP-2 (NP_003245.1) and feline TIMP-2 (XP_023105059.1), showing 95.48% amino acid identity, 100% query coverage and an E-value of 1e-164.

Score	Expect	Method	Identities	Positives	Gaps
441 bits(1133)	1e-164	Compositional matrix adjust.	211/221(95%)	217/221(98%)	1/221(0%)

```
Query  1    MGAAARTLRLALGLLLL-ATLLRPADACSCSPVHPQQAFCNADVVIRAKAVSEKEVDSGN  59
            MG+AAR+LRLALGLLLL  TLLR ADACSCSPVHPQQAFCNADVVIRAKAVS KEVDSGN
Sbjct  1    MGSAARSLRLALGLLLLLGTLLRRADACSCSPVHPQQAFCNADVVIRAKAVSVKEVDSGN  60

Query  60   DIYGNPIKRIQYEIKQIKMFKGPEKDIEFIYTAPSSAVCGVSLDVGGKKEYLIAGKAEGD  119
            DIYGNPIKRIQYEIKQIKMFKGP+KDIEFIYTAPSSAVCGVSLD+GGKKEYLIAGKAEG+
Sbjct  61   DIYGNPIKRIQYEIKQIKMFKGPDKDIEFIYTAPSSAVCGVSLDIGGKKEYLIAGKAEGN  120

Query  120  GKMHITLCDFIVPWDTLSTTQKKSLNHRYQMGCECKITRCPMIPCYISSPDECLWMDWVT  179
            GK+HITLCDFIVPWDTLSTTQKKSLNHRYQMGCECKITRCPMIPCYISSPDECLWMDWVT
Sbjct  121  GKLHITLCDFIVPWDTLSTTQKKSLNHRYQMGCECKITRCPMIPCYISSPDECLWMDWVT  180

Query  180  EKNINGHQAKFFACIKRSDGSCAWYRGAAPPKQEFLDIEDP  220
            EKNINGHQAKFFACIKRSDGSCAWYRGAAPPKQEFLDIEDP
Sbjct  181  EKNINGHQAKFFACIKRSDGSCAWYRGAAPPKQEFLDIEDP  221
```
