# Supplementary material for: Microenvironment of Sarcomas Associated and Not Associated with Inoculation Sites in Domestic Cats: The Role of Metalloproteinases and Angiogenic Factors
Source: Biology (Basel). 2026 Aug 6;15(15):1322. doi: 10.3390/biology15151322 (PMC13464654; doi:10.3390/biology15151322)
Supplement: Supplementary file 1 [file biology-15-01322-s001.zip › Supplementary Material S6.pdf]

Supplementary Material S6. Protein BLAST alignment between human VEGF-A (NP_001303939.1) and feline VEGF-A (NP_001422838.1), showing 83.18% amino acid identity, 100% query coverage and an E-value of 1e-124.

Score	Expect	Method	Identities	Positives	Gaps
346 bits(887)	1e-124	Compositional matrix adjust.	178/214(83%)	183/214(85%)	24/214(11%)

```
Query  1    MNFLLSWVHWSLALLLYLHHAKWSQAAPMAEGGGQNHHEVVKFMDVYQRSYCHPIETLVD  60
            MNFLLSWVHWSLALLLYLHHAKWSQAAPMA+G     HEVVKFMDVYQRSYC PIETLVD
Sbjct  181  MNFLLSWVHWSLALLLYLHHAKWSQAAPMADGE-HKPHEVVKFMDVYQRSYCRPIETLVD  239

Query  61   IFQEYPDEIEYIFKPSCVPLMRCGGCCNDEGLECVPTEESNITMQIMRIKPHQGQHIGEM  120
            IFQEYPDEIEYIFKPSCVPLMRCGGCCNDEGLECVPTEE NITMQIMRIKPHQGQHIGEM
Sbjct  240  IFQEYPDEIEYIFKPSCVPLMRCGGCCNDEGLECVPTEEFNITMQIMRIKPHQGQHIGEM  299

Query  121  SFLQHNKCECRPKKDRARQEN-----------------------PCGPCSERRKHLFVQD  157
            SFLQH+KCECRPKKDRA+++                        PCGPCSERRKHLFVQD
Sbjct  300  SFLQHSKCECRPKKDRAKEKKSVRGKGKGQKRKRKKSRYKSWSVPCGPCSERRKHLFVQD  359

Query  158  PQTCKCSCKNTDSRCKARQLELNERTCRCDKPRR  191
            PQTCKCSCKNTDSRCKARQLELNERTCRCDKPRR
Sbjct  360  PQTCKCSCKNTDSRCKARQLELNERTCRCDKPRR  393
```
