# Supplementary material for: Microenvironment of Sarcomas Associated and Not Associated with Inoculation Sites in Domestic Cats: The Role of Metalloproteinases and Angiogenic Factors
Source: Biology (Basel). 2026 Aug 6;15(15):1322. doi: 10.3390/biology15151322 (PMC13464654; doi:10.3390/biology15151322)
Supplement: Supplementary file 1 [file biology-15-01322-s001.zip › Supplementary Material S7.pdf]

Supplementary Material S7. Protein BLAST alignment between human VEGFR-2 (NP_002244.1) and feline VEGFR-2 (XP_011280181.3), showing 93.29% amino acid identity, 100% query coverage and an E-value of 0.

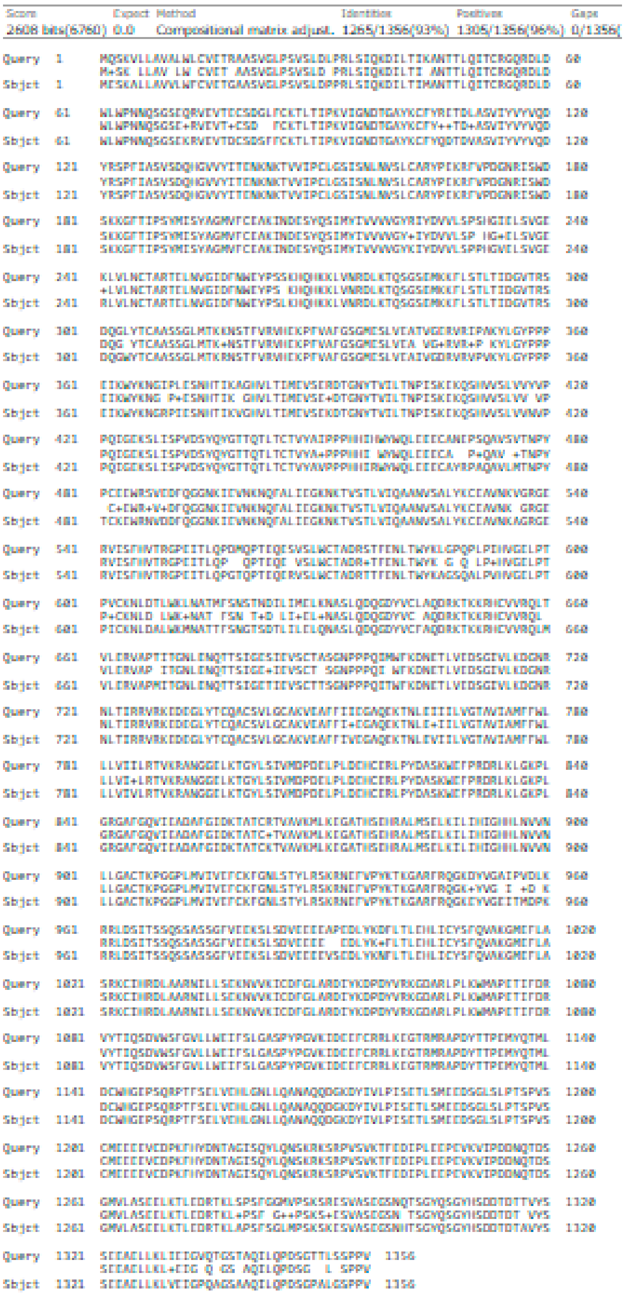

Score	Expect	Method	Identities	Positives	Gaps
2608 bits(6760)	0.0	Compositional matrix adjust.	1265/1356(93%)	1305/1356(96%)	0/1356(
