# Supplementary material for: Microenvironment of Sarcomas Associated and Not Associated with Inoculation Sites in Domestic Cats: The Role of Metalloproteinases and Angiogenic Factors
Source: Biology (Basel). 2026 Aug 6;15(15):1322. doi: 10.3390/biology15151322 (PMC13464654; doi:10.3390/biology15151322)
Supplement: Supplementary file 1 [file biology-15-01322-s001.zip › Supplementary Material S8.pdf]

Supplementary Material S8. Protein BLAST alignment between human FGFR-2 (NP_000132.3) and feline FGFR-2 (XP_044896695.1), showing 97.56% amino acid identity, 100% query coverage and an E-value of 0.

Score	Expect	Method	Identities	Positives	Gaps
1674 bits(4335)	0.0	Compositional matrix adjust.	801/821(98%)	812/821(98%)	1/821(0%)

```
Query  1    MVSWGRFICLVVVTMATLSLARPSFSLVEDTTLEPEEPPTKYQISQPEVYVAAPGESLEV  60
            MVSWGRF+CLVVVTMATL+LARPSF+LVEDTTLEPEEPPTKYQISQPEVYVAAPGESLE+
Sbjct  21   MVSWGRFLCLVVVTMATLALARPSFNLVEDTTLEPEEPPTKYQISQPEVYVAAPGESLEL  80

Query  61   RCLLKDAAVISWTKDGVHLGPNNRTVLIGEYLQIKGATPRDSGLYACTASRTVDSETWYF  120
            RC L+DA +ISWTKDGVHLGPNNRTVLIGEYLQIKGATPRDSGLYACTA+R VDSE  YF
Sbjct  81   RCPLRDATMISWTKDGVHLGPNNRTVLIGEYLQIKGATPRDSGLYACTAARPVDSEAVYF  140

Query  121  MVNVTDAISSGDDEDDTDGAEDFVSENSNNKRAPYWTNTEKMEKRLHAVPAANTVKFRCP  180
            MVNVTDAISSGDDEDDT+G+EDFVSEN NNKRAPYWTNTEKMEKRLHAVPAANTVKFRCP
Sbjct  141  MVNVTDAISSGDDEDDTEGSEDFVSEN-NNKRAPYWTNTEKMEKRLHAVPAANTVKFRCP  199

Query  181  AGGNPMPTMRWLKNGKEFKQEHRIGGYKVRNQHWSLIMESVVPSDKGNYTCVVENEYGSI  240
            AGGNP PTMRWLKNGKEFKQEHRIGGYKVRNQHWSLIMESVVPSDKGNYTCVVENEYGSI
Sbjct  200  AGGNPTPTMRWLKNGKEFKQEHRIGGYKVRNQHWSLIMESVVPSDKGNYTCVVENEYGSI  259

Query  241  NHTYHLDVVERSPHRPILQAGLPANASTVVGGDVEFVCKVYSDAQPHIQWIKHVEKNGSK  300
            NHTYHLDVVERSPHRPILQAGLPANASTVVGGDVEFVCKVYSDAQPHIQWIKHVEKNGSK
Sbjct  260  NHTYHLDVVERSPHRPILQAGLPANASTVVGGDVEFVCKVYSDAQPHIQWIKHVEKNGSK  319

Query  301  YGPDGLPYLKVLKAAGVNTTDKEIEVLYIRNVTFEDAGEYTCLAGNSIGISFHSAWLTVL  360
            YGPDGLPYLKVLKAAGVNTTDKEIEVLYIRNVTFEDAGEYTCLAGNSIGISFHSAWLTVL
Sbjct  320  YGPDGLPYLKVLKAAGVNTTDKEIEVLYIRNVTFEDAGEYTCLAGNSIGISFHSAWLTVL  379

Query  361  PAPGREKEITASPDYLEIAIYCIGVFLIACMVVTVILCRMKNTTKKPDFSSQPAVHKLTK  420
            PAP REKEITASPDYLEIAIYCIGVFLIACMVVTVILCRMK TTKKPDFSSQPAVHKLTK
Sbjct  380  PAPVREKEITASPDYLEIAIYCIGVFLIACMVVTVILCRMKTTTKKPDFSSQPAVHKLTK  439

Query  421  RIPLRRQVTVSAESSSSMNSNTPLVRITTRLSSTADTPMLAGVSEYELPEDPKWEFPRDK  480
            RIPLRRQVTVSAESSSSMNSNTPLVRITTRLSSTADTPMLAGVSEYELPEDPKWEFPRDK
Sbjct  440  RIPLRRQVTVSAESSSSMNSNTPLVRITTRLSSTADTPMLAGVSEYELPEDPKWEFPRDK  499

Query  481  LTLGKPLGEGCFGQVVMAEAVGIDKDKPKEAVTVAVKMLKDDATEKDLSDLVSEMEMMKM  540
            LTLGKPLGEGCFGQVVMAEAVGIDK+KPKEAVTVAVKMLKDDATEKDLSDLVSEMEMMKM
Sbjct  500  LTLGKPLGEGCFGQVVMAEAVGIDKEKPKEAVTVAVKMLKDDATEKDLSDLVSEMEMMKM  559

Query  541  IGKHKNIINLLGACTQDGPLYVIVEYASKGNLREYLRARRPPGMEYSYDINRVPEEQMTF  600
            IGKHKNIINLLGACTQDGPLYVIVEYASKGNLREYLRARRPPGMEYSYDINRVPEEQMTF
Sbjct  560  IGKHKNIINLLGACTQDGPLYVIVEYASKGNLREYLRARRPPGMEYSYDINRVPEEQMTF  619

Query  601  KDLVSCTYQLARGMEYLASQKCIHRDLAARNVLVTENNVMKIADFGLARDINNIDYYKKT  660
            KDLVSCTYQLARGMEYLASQKCIHRDLAARNVLVTENNVMKIADFGLARDINNIDYYKKT
Sbjct  620  KDLVSCTYQLARGMEYLASQKCIHRDLAARNVLVTENNVMKIADFGLARDINNIDYYKKT  679

Query  661  TNGRLPVKWMAPEALFDRVYTHQSDVWSFGVLMWEIFTLGGSPYPGIPVEELFKLLKEGH  720
            TNGRLPVKWMAPEALFDRVYTHQSDVWSFGVLMWEIFTLGGSPYPGIPVEELFKLLKEGH
Sbjct  680  TNGRLPVKWMAPEALFDRVYTHQSDVWSFGVLMWEIFTLGGSPYPGIPVEELFKLLKEGH  739

Query  721  RMDKPANCTNELYMMMRDCWHAVPSQRPTFKQLVEDLDRILTLTTNEEYLDLSQPLEQYS  780
            RMDKPANCTNELYMMMRDCWHAVPSQRPTFKQLVEDLDRILTLTTNEEYLDLSQPLEQYS
Sbjct  740  RMDKPANCTNELYMMMRDCWHAVPSQRPTFKQLVEDLDRILTLTTNEEYLDLSQPLEQYS  799

Query  781  PSYPDTRSSCSSGDDSVFSPDPMPYEPCLPQYPHINGSVKT  821
            PSYPDTRSSCSSGDDSVFSPDPMPYEPCLPQYPH+NGSVKT
Sbjct  800  PSYPDTRSSCSSGDDSVFSPDPMPYEPCLPQYPHVNGSVKT  840
```
