# Supplementary material for: Microenvironment of Sarcomas Associated and Not Associated with Inoculation Sites in Domestic Cats: The Role of Metalloproteinases and Angiogenic Factors
Source: Biology (Basel). 2026 Aug 6;15(15):1322. doi: 10.3390/biology15151322 (PMC13464654; doi:10.3390/biology15151322)
Supplement: Supplementary file 1 [file biology-15-01322-s001.zip › Supplementary Material S9.pdf]

Supplementary Material S9. Protein BLAST alignment between human CD-31 (NP_000433.4) and feline CD-31 (XP_019673673.2), showing 70.77% amino acid identity, 100% query coverage and an E-value of 0.

Score	Expect	Method	Identities	Positives	Gaps
1061 bits(2743)	0.0	Compositional matrix adjust.	523/739(71%)	609/739(82%)	22/739(2%)

```
Query  1    MQPRWAQGATMWLGVLLTLLLCSSLEGQENSFTINSVDMKSLPDWTVQNGKNLTLQCFAD  60
            MQP  AQ   MWLGVLL L+LC  LEGQEN FTIN + M  LP   VQNG+NLTLQC  D
Sbjct  1    MQPETAQDGKMWLGVLLALMLCPGLEGQENPFTINYIHMNILPGLEVQNGENLTLQCVVD  60

Query  61   VSTTSHVKPQHQMLFYKDDVLFYNISSMKSTESYFIPEVRIYDSGTYKCTVIVNNKEKTT  120
            +STTSHVKPQH +LFYKDDVLF+N+SSMK+TESYFIP+ R+ D+GTYKCTVI+NNKEKTT
Sbjct  61   ISTTSHVKPQHWVLFYKDDVLFHNVSSMKNTESYFIPQARVCDAGTYKCTVILNNKEKTT  120

Query  121  AEYQVLVEGVPSPRVTLDKKEAIQGGIVRVNCSVPEEKAPIHFTIEKLELNEKMVKLKRE  180
            +EYQV V+GV  PRVTLDKKEAI+GG+V VNCSVPEE  PI+FTIEKL+L+ K  K KRE
Sbjct  121  SEYQVWVKGVSDPRVTLDKKEAIEGGVVTVNCSVPEENPPIYFTIEKLKLDAKGFKQKRE  180

Query  181  KNSRDQNFVILEFPVEEQDRVLSFRCQARIISGIHMQTSESTKSELVTVTESFSTPKFHI  240
            K S++QNF+++EF VEEQD V+ F+CQARI SG +++ S++ +SELVTVTESFS P+FHI
Sbjct  181  KVSQNQNFMVMEFTVEEQDHVILFQCQARIFSGTNVEISKAIRSELVTVTESFSNPRFHI  240

Query  241  SPTGMIMEGAQLHIKCTIQVTHLAQEFPEIIIQKDKAIVAHNRHGNKAVYSVMAMVEHSG  300
            SP G+I EG QL+IKCTIQVTHL Q FPEIIIQKDKAIVAH  HGN+A YSVMAMVEHSG
Sbjct  241  SPKGVITEGDQLNIKCTIQVTHLVQSFPEIIIQKDKAIVAHKTHGNEATYSVMAMVEHSG  300

Query  301  NYTCKVESSRISKVSSIVVNITELFSKPELESSFTHLDQGERLNLSCSIPGAPPANFTIQ  360
            NYTCKVE+SRISKVSSIVVNITELFSKP+LESS T LD GE LNL CSIPGAPPANFTIQ
Sbjct  301  NYTCKVEASRISKVSSIVVNITELFSKPKLESSATRLDHGESLNLWCSIPGAPPANFTIQ  360

Query  361  KEDTIVSQTQDFTKIASKSDSGTYICTAGIDKVVKKSNTVQIVVCEMLSQPRISYDAQFE  420
            KE+TIVS +Q+FTKIAS+ D GTY C AGI KVVKKSN VQI VCEMLS+PRI YD+  E
Sbjct  361  KENTIVSLSQNFTKIASERDRGTYTCNAGIGKVVKKSNAVQITVCEMLSKPRIFYDSGPE  420

Query  421  VIKGQTIEVRCESISGTLPISYQLLKTSKVLENSTKNSNDPAVFKDNPTEDVEYQCVADN  480
            VIKGQTI V C+S++GT PISY L+K + +LE+   +SN+PAVFKD PT+DVEYQC+ADN
Sbjct  421  VIKGQTIAVSCQSVNGTAPISYHLVKANSILESCNMDSNEPAVFKDKPTKDVEYQCIADN  480

Query  481  CHSHAKMLSEVLRVKVIAPVDEVQISILSSKVVESGEDIVLQCAVNEGSGPITYKFYREK  540
            CHSH +M+SEVLRVKVIAPVD+V+++IL ++ VESG+++VL+C+VN+ SGPITY+FYREK
Sbjct  481  CHSHTEMISEVLRVKVIAPVDQVKLTILLNEEVESGKEVVLRCSVNDASGPITYRFYREK  540

Query  541  EGKPFYQMTSNATQAFWTKQKASKEQEGEYYCTAFNRANHASSVPRSKILTVRVILAPWK  600
            EG PFYQ+ SN T A W K KASKE EG+YYCTAFNRAN   S P+S ILTVRV LAPWK
Sbjct  541  EGSPFYQIISNDTYAIWHKDKASKEHEGQYYCTAFNRANLVKSNPQSNILTVRVFLAPWK  600

Query  601  KGLIAVVIIGVIIALLIIAAKCYFLRKAKAKQMPVEMSRPAVPLLNSNNEKM-SDPNMEA  659
            KGLIAVV+IGVIIA LI+ A+C FL+KAKAKQ PVEM+RPA PLLNS+NEKM SDP+ EA
Sbjct  601  KGLIAVVVIGVIIATLILGARCCFLKKAKAKQTPVEMARPAAPLLNSSNEKMLSDPDTEA  660

Query  660  NSHYGHNDDVRNHAMKPINDNKEPLNSDVQYTEVQVSSAESHKDLGKKDTETVYSEVRKA  719
            N HYG+++DV NHAMKP N+N                     K L  K TETVYSE+RK
Sbjct  661  NRHYGYSEDVGNHAMKPANEN---------------------KGLETKGTETVYSEIRKT  699

Query  720  VPDAVESRYSRTEGSLDGT  738
             PD VE+RYSRTEGSLDGT
Sbjct  700  NPDFVENRYSRTEGSLDGT  718
```
